# Role of Fatty Acid Kinase in Cellular Lipid Homeostasis and SaeRS-Dependent Virulence Factor Expression in *Staphylococcus aureus*

**DOI:** 10.1128/mBio.00988-17

**Published:** 2017-08-01

**Authors:** Megan E. Ericson, Chitra Subramanian, Matthew W. Frank, Charles O. Rock

**Affiliations:** Department of Infectious Diseases, St. Jude Children’s Research Hospital, Memphis, Tennessee, USA; University of Washington

**Keywords:** Staphylococcus aureus, fatty acid, hemolysin, phospholipid, two-component regulatory systems, virulence factors, virulence regulation

## Abstract

The SaeRS two-component system is a master activator of virulence factor transcription in *Staphylococcus aureus*, but the cellular factors that control its activity are unknown. Fatty acid (FA) kinase is a two-component enzyme system required for extracellular FA uptake and SaeRS activity. Here, we demonstrate the existence of an intracellular nonesterified FA pool in *S. aureus* that is elevated in strains lacking FA kinase activity. SaeRS-mediated transcription is restored in FA kinase-negative strains when the intracellular FA pool is reduced either by growth with FA-depleted bovine serum albumin to extract the FA into the medium or by the heterologous expression of *Neisseria gonorrhoeae* acyl-acyl carrier protein synthetase to activate FA for phospholipid synthesis. These data show that FAs act as negative regulators of SaeRS signaling, and FA kinase activates SaeRS-dependent virulence factor production by lowering inhibitory FA levels. Thus, FA kinase plays a role in cellular lipid homeostasis by activating FA for incorporation into phospholipid, and it indirectly regulates SaeRS signaling by maintaining a low intracellular FA pool.

## IMPORTANCE

The SaeRS two-component system is a master transcriptional activator of virulence factor production in response to the host environment in *S. aureus*, and strains lacking FA kinase have severely attenuated SaeRS-dependent virulence factor transcription. FA kinase is required for the activation of exogenous FAs, and it plays a role in cellular lipid homeostasis by recycling cellular FAs into the phospholipid biosynthetic pathway. Activation of the sensor kinase, SaeS, is mediated by its membrane anchor domain, and the FAs which accumulate in FA kinase knockout strains are potent inhibitors of SaeS-dependent signaling. This work identifies FAs as physiological effectors for the SaeRS system and reveals a connection between cellular lipid homeostasis and the regulation of virulence factor transcription. FA kinase is widely distributed in Gram-positive bacteria, suggesting similar roles for FA kinase in these organisms.

## INTRODUCTION

Methicillin-resistant *Staphylococcus aureus* (MRSA) continues to be a leading cause of soft tissue infections such as cellulitis and more serious infections, like endocarditis and sepsis (1, 2). Secreted proteins known as virulence factors contribute to the tissue damage, nutrient acquisition, and immune evasion to accelerate the spread of infection (3). Virulence factor transcription in *S. aureus* is governed by a complex regulatory network that includes alternate sigma factor B, the DNA-binding protein SarA, the *agr* quorum-sensing pathway, and the SaeRS two-component system (4, 5). This study focuses on the SaeRS system, which consists of a membrane-bound sensor kinase, SaeS, and a soluble DNA-binding response regulator, SaeR (6). The SaeS sensor kinase belongs to a family of histidine kinases that are anchored to the membrane by two transmembrane helices connected by a 9-amino-acid extracellular loop that lacks a globular ligand binding domain (7–9). Activated SaeS autophosphorylates on a conserved histidine residue, and the phosphate is then transferred to a conserved aspartate on SaeR to trigger DNA binding and transcriptional activation (6). Phosphorylated SaeR activates the expression of over 20 virulence factor genes, including those for α-hemolysin toxin (*hla*), extracellular fibrinogen binding protein (*efb*), and the immune evasion proteins Ehp (*ehp*) and Sbi (*sbi*) (10–12). SaeRS-null bacteria are less infective in animal models, highlighting the importance of this system in pathogenesis (13, 14). SaeS signaling increases following exposure to human neutrophil defensins, calprotectin, and hydrogen peroxide (15–18). Conversely, silkworm apolipophorin protein, acidic pH, AFN-1252, cerulenin, fatty acids (FA), and linezolid all inhibit SaeRS activity (19–25). Site-directed mutagenesis has illustrated that the conformation of the membrane anchor domain controls SaeS sensor kinase activity (26, 27). Downstream signaling by bacterial sensor kinases is controlled by changes in the conformation of the helical transmembrane anchor domains (28). It remains to be determined how the mutations in the membrane anchor domain alter SaeS structure and sensing.

FA kinase is a two-component enzyme consisting of a kinase domain protein (FakA) that phosphorylates an FA bound to a FA binding protein (FakB) (29, 30). FakB transfers the acyl-phosphate to PlsX to be converted to acyl-acyl carrier protein (ACP) and enter the fatty acid biosynthetic cycle or to PlsY for the acylation of glycerol phosphate (31, 32). FA kinase is the only pathway for FA activation and incorporation into *S. aureus* membrane phospholipids (29, 33). A FA kinase-null *S. aureus* strain was resistant to dermcidin (34) and exhibits increased biofilm formation (35). However, the most striking phenotype of FA kinase knockout strains is the lack of α-hemolysin production, indicating a novel role for FA kinase in the control of virulence factor production (36). A genome-wide analysis showed that FA kinase-null strains were specifically deficient in the expression of all virulence factors controlled by the SaeRS system (29). The fact that acetyl-phosphates are known to phosphorylate response regulators suggests that FA kinase may participate in the regulatory phosphorylation cascade in the SaeRS system (29, 37). Although it is clear that transcription of the SaeRS virulence regulon is supported by a functional FA kinase, the connection between FA kinase and SaeRS has not been established.

The goal of this study was to establish a biochemical connection between FA kinase and the activity of the SaeRS system. We found that FA kinase activation of virulence factor transcription requires the SaeRS two-component system, but FA kinase does not directly phosphorylate either SaeS or SaeR. Instead, FA are inhibitors of SaeS phosphorylation of SaeR and accumulate in FA kinase-null bacteria. FA removal by growth with bovine serum albumin (BSA) or by the ectopic expression of an acyl-ACP synthetase restored transcription of the *saePQRS* operon and downstream SaeRS-regulated genes, showing that it is FA rather than FA kinase that regulates SaeRS signaling. Thus, the 10-fold downregulation of SaeRS signaling in FA kinase-null cells is due to the accumulation of cellular FA, which in turn negatively regulates SaeRS signaling.

## RESULTS

### FA kinase impact on virulence factor transcription requires SaeRS.

**FIG 1:**
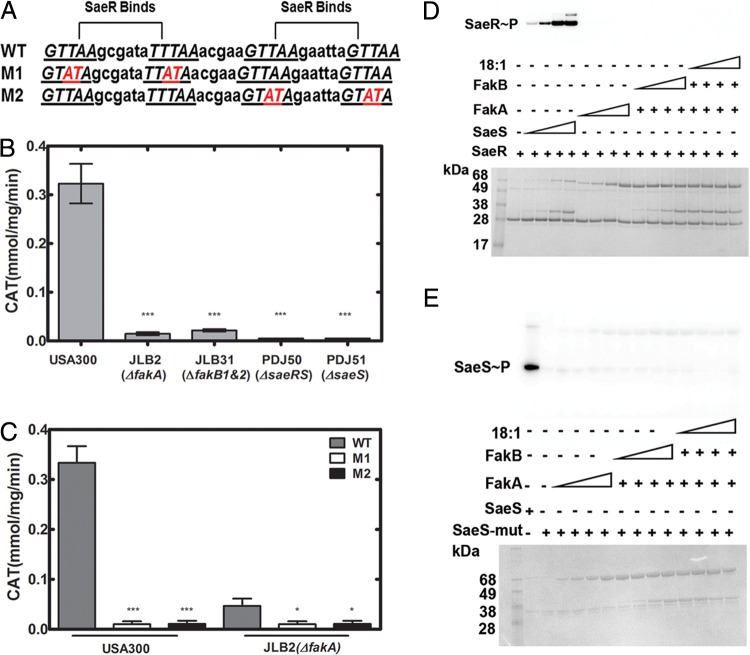
FA kinase does not act directly on SaeRS. (A) Diagram of the two SaeR binding sites within the 145-bp *saePQRS* promoter. The promoter was fused to the coding sequence of chloramphenicol acetyltransferase to provide a readout of SaeRS activity. Two mutant promoter constructs, M1 and M2, each containing one wild-type and one mutated SaeR binding site (highlighted in red), were generated. (B) Transcriptional activity of the wild-type *saePQRS* promoter in strain USA300 and derivatives JLB2 (Δ*fakA* mutant), JLB31 (Δ*fakB1&2* mutant), PDJ50 (Δ*saeS* mutant), and PDJ51 (Δ*saeRS* mutant). Cultures were grown to an OD_600_ of 2 before bacteria were harvested and CAT activity in cell lysates was measured. Data shown are from three biological replicates. (C) Transcriptional activity of the wild-type or mutant *saePQRS* promoters in strains USA300 and JLB2 (Δ*fakA*). Data shown are from three biological replicates. (D) FA kinase phosphorylation of SaeR was analyzed in reaction mixtures containing [γ-^32^P]ATP followed by gel electrophoresis. SaeR was incubated with 1, 2, 5, or 10 μM SaeS as a positive control. The other SaeR phosphorylation reaction mixtures contained 1, 2, 5, or 10 μM FakA, 1, 2, 5, or 10 μM FakB (with 18:1) in the presence of 10 μM FakA, or 10, 20 40, or 80 μM oleic acid (18:1) in the presence of 10 μM FakB (18:1) and 10 μM FakA. (E) SaeS phosphorylation by FA kinase was analyzed in reaction mixtures containing [γ-^32^P]ATP and SaeS(K252A R298A) (SaeS-mut). SaeS-mut has two missense mutations in the ATP binding domain to prevent autophosphorylation (39). Wild-type SaeS autophosphorylation was the positive control. Phosphorylation reaction mixtures contained 1, 2, 5, or 10 μM FakA, 1, 2, 5, or 10 μM FakB2 (18:1) plus 10 μM FakA, or 10, 20 40, or 80 μM oleic acid (18:1) in the presence of 10 μM FakB and 10 μM FakA. In panels D and E, the gel images on top are the autoradiograms and the gel images at the bottom are the stained protein gel. Significance was determined by the two-tailed Student *t* test. *, *P* < 0.05; **, *P* < 0.01; ***, *P* < 0.001.

We first confirmed that FA kinase is dependent upon SaeRS to influence virulence factor transcription. A reporter construct to monitor the activity of the SaeR-controlled *saePQRS* promoter was created by fusing the *saePQRS* promoter to the chloramphenicol acetyltransferase (CAT) coding sequence to identify the DNA sequences required for FA kinase activation of the promoter (Fig. 1A). Accordingly, robust transcription of the reporter was observed in the wild-type strain USA300 but was absent in strains lacking SaeRS (strain PDJ50) or SaeS (strain PDJ51) (Fig. 1B). FA kinase inactivation significantly depressed transcription from the *saePQRS* promoter, showing that the putative FA kinase regulatory elements are located within the promoter construct (Fig. 1B). Activation of the *saePQRS* promoter requires both SaeR binding sites (11); therefore, we created promoter mutants that inactivated one or the other of the SaeR sites (Fig. 1A). Neither of these mutant promoter constructs was active in the wild-type USA300 or the FA kinase knockout (JLB2) strains (Fig. 1C). These data showed that FA kinase regulation of *saePQRS* expression requires both SaeR binding sites, suggesting that FA kinase modifies SaeRS signaling rather than functioning independently on the promoter.

### FA kinase does not phosphorylate SaeS or SaeR.

The SaeRS two-component system is activated via phosphorylation (6). Response regulators in Gram-negative two-component systems can accept phosphate from acetyl-phosphate (38), leading to the hypothesis that acyl-phosphate produced by FA kinase phosphorylates either SaeS or SaeR (38). FakA, FakB2, SaeS, and SaeR proteins were expressed, purified, and used in reactions with [^32^P]ATP to examine phosphotransfer from FA kinase to the SaeRS system. We first tested phosphorylation of SaeR. SaeS phosphotransfer to SaeR was included as a positive control (Fig. 1D). FakA was unable to phosphorylate SaeR when added at concentrations from 1 μM to 10 μM (Fig. 1D). Introducing FakB2 into the reaction mix did not result in SaeR phosphorylation (Fig. 1D). Oleic acid (18:1) was included to drive the production of acyl-phosphate, but its presence did not lead to phosphorylated SaeR (Fig. 1D). We next examined phosphotransfer from FA kinase to SaeS. Two residues in the ATP binding region of SaeS (K252A R298A) (39) were mutated to ensure that the ^32^P signal for SaeS was transferred from FA kinase and not the results of autophosphorylation. Wild-type SaeS was used as a positive control (Fig. 1E). The SaeS mutant displayed no autophosphorylation activity (Fig. 1E). Addition of FakA with the SaeS mutant did not result in SaeS phosphorylation (Fig. 1E). The same result occurred with the addition of FakB2 and oleic acid (18:1) (Fig. 1E). Acyl-phosphate produced by FakA and FakB was not detectable on SDS-PAGE gels, but it was quantified using ^14^C-labeled FA as the substrate to produce [^14^C]acyl-phosphate (29). A FA kinase assay performed in parallel showed the active formation of acyl-phosphate (specific activity, 6 μmol/min/mg).

### Addition of exogenous FA inhibits SaeRS signaling.

**FIG 2:**
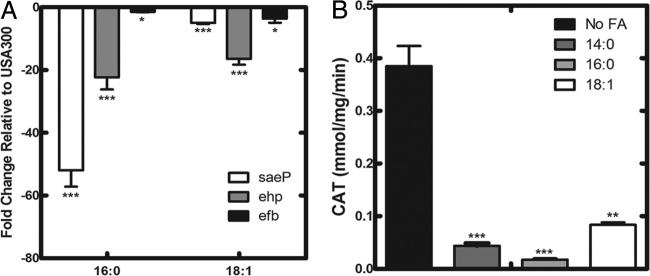
Exogenous FA inhibits SaeRS activity. (A) Transcript levels of SaeRS-regulated genes after the addition of FA. Overnight cultures of USA300 were diluted and grown to an OD_600_ of 0.5 before 500 μM FA and 10 mg/ml BSA were added for 2 h. Fold changes shown are in relation to results with a USA300 strain grown with only 10 mg/ml BSA. The data are from two biological replicates that were each analyzed in triplicate. (B) *saePQRS* promoter activity in USA300 grown in the absence or presence of 500 μM myristate (14:0), palmitate (16:0), or oleate (18:1) with 10 mg/ml of FA-free BSA. Cultures were grown to an OD_600_ of 2 before bacteria were harvested and CAT activity in cell lysates was measured. Data are from three biological replicates. Significance was determined by the two-tailed Student *t* test. *, *P* < 0.05; **, *P* < 0.01; ***, *P* < 0.001.

An analysis of the global transcriptional response to oleic acid (18:1) addition to *S. aureus* cultures showed that FA strongly inhibit SaeRS signaling (25). To test if FA inhibition of SaeRS in USA300 bacteria was indeed limited to the 18:1 FA species, we added 500 μM of either 16:0 (palmitate) or 18:1 in the presence of 10 mg/ml FA-free BSA to USA300 bacteria. No growth differences were noted between bacteria grown with versus without exogenous FA (40). Cells were harvested, and RNA was isolated for quantitative real-time PCR (qRT-PCR). Three representative genes controlled by SaeRS were downregulated by the addition of either of the FA species (Fig. 2A). To further confirm the inhibitory effect of exogenous FA, we added 500 μM myristate (14:0), 16:0, or 18:1 in the presence of 10 mg/ml BSA to strain USA300 harboring the CAT reporter plasmid. Transcriptional activity of the s*aePQRS* promoter was downregulated with the addition of each FA (Fig. 2B). BSA is necessary to present the FA to the cells and prevent the toxic effects of the 14:0 FA on the bacteria (40). Krute et al. (37) reported that 14:0 did not alter SaeRS signaling in strain USA300, but that study did not employ BSA to present the FA and avoid toxicity.

### Nonesterified FA accumulate in FA kinase-null strains.

**FIG 3:**
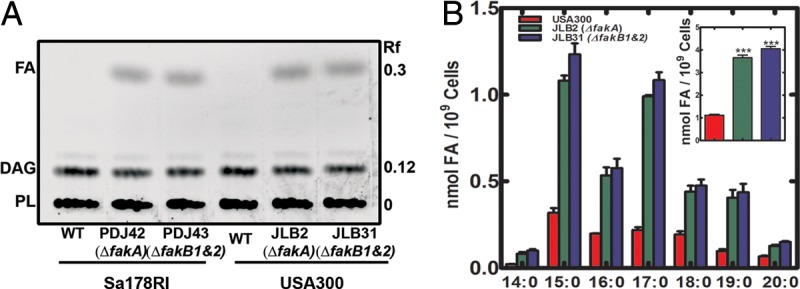
FA accumulated in FA kinase-null backgrounds of two strains. (A) Strains Sa178RI and USA300 and their Δ*fakA* and Δ*fakB1&2* mutant derivatives were metabolically labeled with [^14^C]acetic acid for 3 h, and the lipids were extracted and fractionated by thin-layer chromatography. The chromatogram is representative of the results of three experiments. PL, phospholipid; DAG, diacylglycerol. (B) FA levels in strains USA300, JLB2 (Δ*fakA* mutant), and JLB31 (Δ*fakB1&2* mutant) grown to an OD_600_ of 2 to 3 followed by lipid extraction and quantitative mass spectrometry to determine the amounts of each FA in the strain. The inset shows the effect of FA kinase inactivation on the total FA pool size. The data are from three biological replicates. Significance was determined by the two-tailed Student *t* test. *, *P* < 0.05; **, *P* < 0.01; ***, *P* < 0.001.

FA are the substrates for FA kinase; therefore, we determined whether cellular FA accumulated in strains lacking the kinase. Sa178RI and USA300 strains were metabolically labeled with [^14^C]acetic acid for 3 h, and lipids were extracted and analyzed by thin-layer chromatography (TLC). Phospholipid and diacylglycerol were the most abundant lipids in all strains. However, a ^14^C-labeled nonesterifed FA band was detected in both Δ*fakA* mutant (strains PDJ42 and JLB2) and Δ*fakB1&B2* mutant (strains PDJ43 and JLB31) backgrounds (Fig. 3A) but appeared to be absent in the wild-type strains. Mass spectrometry was used to quantify the amount of free FA in the strains. FA was detected in wild-type strains, illustrating that FA are a normal, albeit minor, component of the normal cellular lipidome (Fig. 3B). As shown by the metabolic labeling experiment, FA accumulated in the FA kinase-null background strains to become a more significant component of the cellular lipidome (Fig. 3B).

### FA, rather than FA kinase, regulates SaeRS activity.

**FIG 4:**
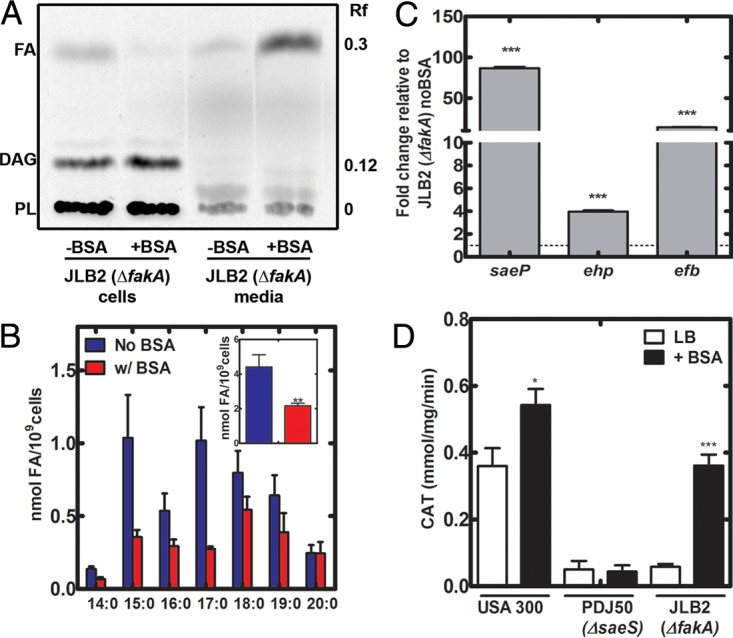
BSA removes FA and restores virulence factor signaling. (A) FA-deficient BSA extracts cellular FA from strain JBL2 (Δ*fakA* mutant). Strain JLB2 (Δ*fakA*) was metabolically labeled with [^14^C]acetic acid and grown in the presence or absence of 10 mg/ml FA-deficient BSA for 3 h, beginning at an OD_600_ of 0.05. Lipids were extracted from the cells and media and fractionated on Silica G layers. The chromatogram shown is representative of 3 biological replicates. PL, phospholipid; DAG, diacylglycerol. (B) Quantification of FA levels in strain JLB2 (Δ*fakA*) grown with or without FA-deficient BSA to an OD_600_ of 2.5 before lipids were extracted and analyzed by mass spectrometry. The inset compares the total cellular FA pool size after growth with or without BSA. Data are from 6 biological replicates. (C) Transcript levels of SaeR regulated genes in strain JLB2 (Δ*fakA*) with BSA. Overnight cultures of strain JLB2 were diluted to an OD_600_ of 0.05 and grown with or without BSA to an OD_600_ of 2 before the cells were harvested and RNA was extracted. qRT-PCR was performed on three SaeR-regulated genes to determine the fold change caused by the addition of FA-deficient BSA. Data are from two biological replicates performed in triplicate. (D) *saePQRS* promoter activity (CAT assay) in strain USA300 and its Δ*saeRS* and Δ*fakA* mutant derivatives carrying the reporter plasmid were grown in the presence or absence of FA-deficient BSA. Data are from four biological replicates.

If the accumulation of cellular FA in FA kinase knockout strains inhibits SaeS signaling, reducing the cellular FA should activate SaeRS. To test this, we added FA-deficient BSA to the growth medium (10 mg/ml) to remove FA from the cells by providing an extracellular sink for these lipids. Strain JLB2 (Δ*fakA*) was grown with or without the addition of BSA and metabolically labeled with [^14^C]acetate. TLC analysis showed that BSA extracted a significant portion of the intracellular FA into the medium, while FA remained predominantly cell associated without addition of BSA (Fig. 4A). Extension of these experiments to strains USA300 and JLB31 (Δ*fakB1&B2*) confirmed that BSA extracts FA from all strains (see Fig. S1 in the supplemental material). Quantitative mass spectrometry showed a 50% decrease of cellular FA levels in strain JLB2 (Δ*fakA*) grown in BSA compared to growth without BSA (Fig. 4B, inset). qRT-PCR showed that three SaeR-regulated transcripts were elevated by the removal of FA from strain JLB2 (Δ*fakA*) by growth with BSA (Fig. 4C). This result was corroborated by an increase in *saePQRS* promoter activity from the reporter plasmid in strain JLB2 (Δ*fakA*) grown with BSA (Fig. 4D). Likewise, BSA increased activity of the *saePQRS* promoter in a USA300 background, demonstrating that levels of FA in the wild-type strain USA300 background are inhibitory to SaeRS signaling (Fig. 4D). BSA had no effect on promoter activity in SaeS knockouts (Fig. 4D).

10.1128/mBio.00988-17.1 FIG S1  BSA removes FA from wild-type and FA kinase-null strains. Strains USA300, JLB2 (Δ*fakA* mutant), and JLB31 (Δ*fakB1* & *2* mutant) were metabolically labeled with [^14^C]acetic acid and grown in the presence or absence of 10 mg/ml FA-deficient BSA for 3 h, beginning at an OD_600_ of 0.05. Lipids were extracted from the cells and media and fractionated on Silica G layers. The image is representative of results from two experiments. DAG, diacylglycerol; PL, phospholipid. Download FIG S1, TIF file, 0.2 MB. Copyright © 2017 Ericson et al. 2017 Ericson et al. This content is distributed under the terms of the Creative Commons Attribution 4.0 International license.

**FIG 5:**
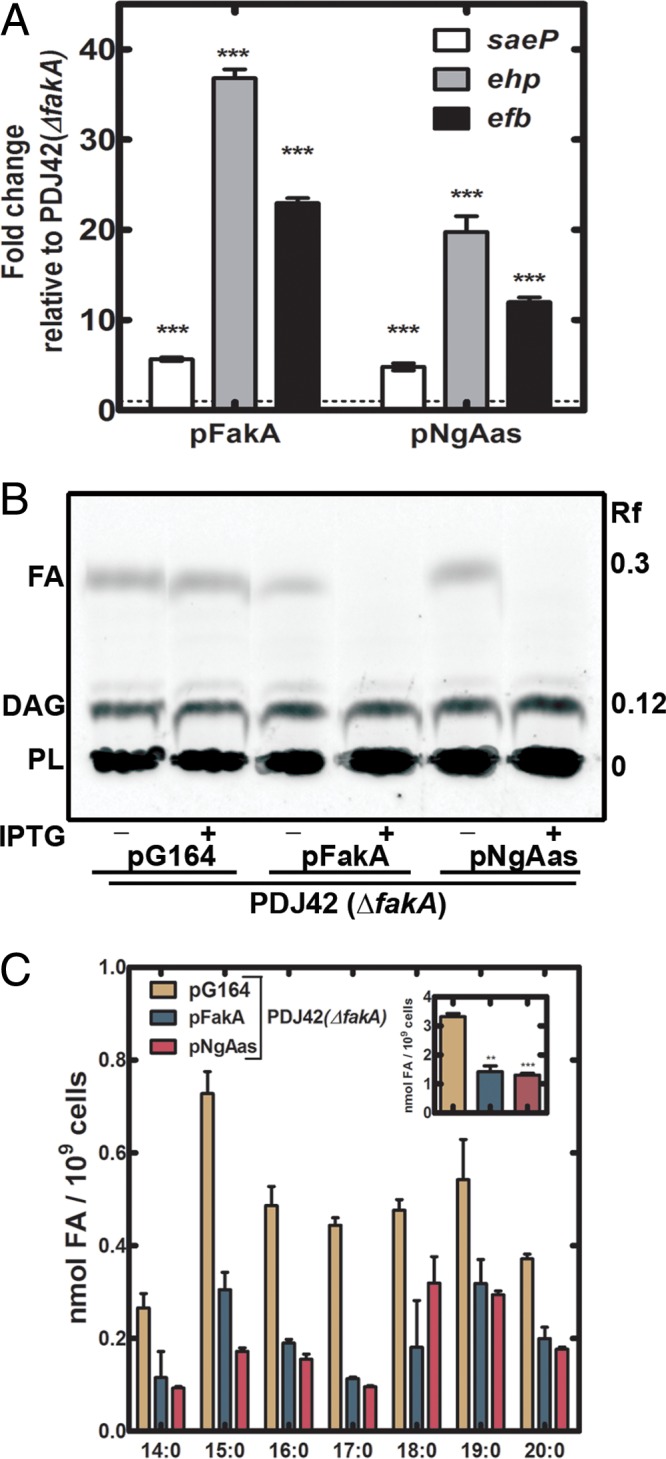
Reduction of cellular FA and activation of virulence factor transcription by NgAas expression in strain Sa178RI. (A) Transcript levels of three selected SaeRS-regulated genes following the introduction of expression vectors pFakA(pPJ497) and pNgAas(pJD001) into a strain Sa178RI Δ*fakA* background. Bacteria were grown with IPTG and harvested upon reaching an OD_600_ of 2. RNA was extracted, and quantitative analysis of SaeR-regulated transcripts by qRT-PCR was performed. Data were from two biological replicates performed in triplicate. (B) FA levels in strain PDJ42 (Δ*fakA*) in the presence and absence of plasmid-driven expression of either FakA or NgAas. Strains were grown with or without 100 μM IPTG to induce expression of FakA or NgAas and labeled with [^14^C]acetic acid for 3 h. Bacteria were harvested and lipids were fractionated on Silica G gel layers. PL, phospholipid; DAG, diacylglycerol. The chromatogram shown is representative of results with 3 biological replicates. (C) FA levels measured by quantitative mass spectrometry. Overnight cultures were diluted and grown to an OD_600 _of 0.5 before addition of 100 μM IPTG. Bacteria were harvested 2 h after IPTG addition and lipids were extracted. Data shown are from three biological replicates. Significance was determined by the two-tailed Student *t* test. *, *P* < 0.05; **, *P* < 0.01; ***, *P* < 0.001.

We next reduced cellular FA levels by the heterologous expression of a protein that would activate cellular FA for incorporation into phospholipids. *Neisseria gonorrhoeae* acyl-ACP synthetase (NgAas) (41) was cloned into the isopropyl-β-d-thiogalactopyranoside (IPTG)-regulated vector pG164 and expressed in strain PDJ42 (Δ*fakA*) derived from strain Sa178R1. Three sentinel SaeRS-regulated virulence factor transcripts were elevated in strain PDJ42 (Δ*fakA*) by the expression of either NgAas or FakA, illustrating that NgAas expression complemented the FA kinase defect in virulence factor transcription (Fig. 5A). [^14^C]acetate metabolic labeling and TLC demonstrated that expression of both NgAas and FakA significantly reduced the levels of intracellular FA in strain PDJ42 (Δ*fakA*) (Fig. 5B). Finally, quantitative mass spectrometry confirmed that NgAas or FakA expressed in strain PDJ42 (Δ*fakA*) reduced cellular FA (Fig. 5C). The restoration of SaeRS signaling by NgAas expression leads to the conclusion that FA, rather than an activity of FA kinase itself, are responsible for the inhibition of SaeRS-dependent virulence factor transcription in Δ*fakA* strains.

### Increased FakA expression decreases FA and increases transcription in a wild-type strain.

**FIG 6:**
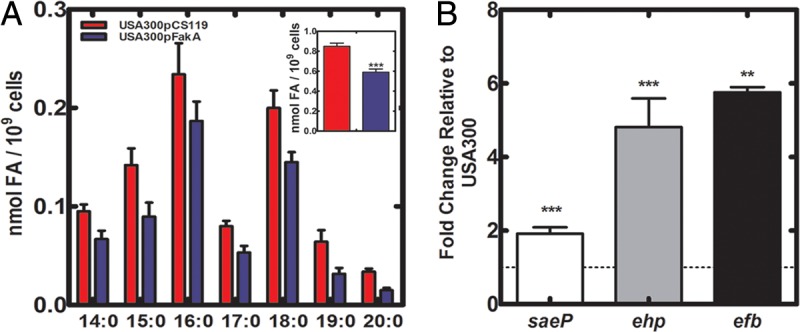
Overexpression of FA kinase lowers FA levels and increases SaeRS activity. (A) Cellular FA levels in strains USA300/pCS119 (empty vector) and USA300/pJLB165(FakA) were determined by quantitative mass spectrometry. The inset shows the comparison of the FA pool size in the two strains. Data are from 6 biological replicates. (B) Levels of three representative SaeRS-regulated transcripts in strains overexpressing FakA. Overnight cultures of strains USA300/pCS119 (empty vector) and USA300/pJLB165(FakA) were diluted and grown to an OD_600_ of 2, cells were harvested, RNA was extracted, and the levels of the indicated transcripts were determined by qRT-PCR. The data are from two independent experiments performed in triplicate. Significance was determined by using the two-tailed Student *t* test. *, *P* < 0.05; **, *P* < 0.01; ***, *P* < 0.001.

Inclusion of FA-deficient BSA in the medium increased *saePQRS* transcription in strain USA300 (Fig. 4D), highlighting that FA levels in the wild-type background restrain SaeRS activity. We corroborated this conclusion by examining the impact of elevated expression of FakA in a wild-type strain USA300 on FA levels and transcription of SaeRS-regulated genes. Quantitative mass spectrometry showed that the plasmid-driven expression of FakA in strain USA300 reduced the intracellular FA pool (Fig. 6A, inset). Furthermore, the expression levels of *saeP*, *ehp*, and *efb* genes were elevated in FakA-overexpressing cells (Fig. 6B). These data confirmed the role of FA kinase in recycling FA into phospholipids and showed that intracellular FA in *S. aureus* functionally represses SaeRS signaling in wild-type strains.

### Hemolytic activity is regulated by cellular FA levels.

**FIG 7:**
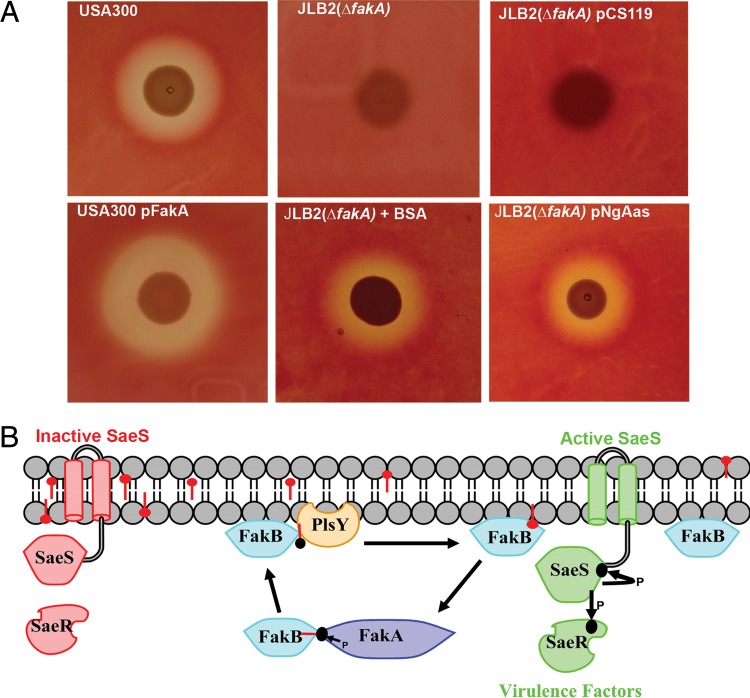
Integration of membrane lipid homeostasis and SaeRS activity. (A) Cellular FA inhibits production of α-hemolysin, as assessed by the diameter of the cleared zones on blood agar plates. α-Hemolysin production in parent strain USA300 was increased by expression of FakA (USA300/pFakA). α-Hemolysin production was not detected in the strain USA300 Δ*fakA* derivative or in the Δ*fakA* mutant strain harboring the empty expression vector [JLB2(ΔfakA)/pCS119]. Inclusion of BSA in the medium [JLB2(ΔfakA) + BSA)] or expression of the *Neisseria* acyl-ACP synthetase [JLB2(ΔfakA)/pNGAas] restored α-hemolysin production to that of a Δ*fakA* strain. The photos are representative of results from multiple experiments. (B) FA metabolism by FA kinase. The basal intracellular membrane FA pool (depicted by red ball-and-stick symbols) in *S. aureus* arises from an unknown metabolic process and is elevated by exposure to exogenous FA. FA partition into the phospholipid bilayer, where they inhibit SaeS sensor kinase activity and virulence factor transcription. The catalytic cycle of the two-component FA kinase system recycles cellular FA into the FASII and phospholipid biosynthetic pathways. FA are picked up by FakB, phosphorylated by FakA, and reincorporated into phospholipid via either PlsX or PlsY. The inactivation of FA kinase (either FakA or FakB) leads to accumulation of FA. Active SaeS is shown in green, and inactive SaeS is shown in red. FA∼P, acyl-phosphate; PlsX, FA∼P:ACP transacylase; PlsY, FA~P-dependent glycerol phosphate acyltransferase; PlsC, acyl-ACP-dependent 1-acylglycerol-phosphate acyltransferase; FASII, bacterial type II FA biosynthesis system.

The elimination of α-hemolysin production was the first recognized phenotype of Δ*fakA* mutant strains (36). α-Hemolysin is regulated by SaeRS (42), and its expression can be monitored by measuring zones of clearance produced by bacteria on a blood agar plate. Plasmid-driven expression of FakA in strain USA300 increased production of α-hemolysin (Fig. 7A), consistent with the lowering of cellular FA in this strain (Fig. 6A). As expected, α-hemolysin production was almost eliminated in strain JLB2 (Δ*fakA*) compared to its wild-type counterpart, strain USA300 (Fig. 7A). α-Hemolysin production was restored in JLB2 (Δ*fakA*) either by growth on blood agar plates supplemented with FA-deficient BSA or by the expression of NgAas (Fig. 7A); these findings are also consistent with the reduction of FA and the transcriptional activation of other SaeRS targets by these treatments (Fig. 4 and 5). The restoration of α-hemolysin production independent of FA kinase function supports the role of cellular FA in inhibiting SaeRS-dependent transcription.

## DISCUSSION

Our results lead to a mechanistic model for the roles of FA kinase in cellular lipid homeostasis in *S. aureus* (Fig. 7B). One function is to recycle FA into the phospholipid biosynthetic pathway. FA recycling begins when FakB binds to FA that spontaneously partition into the phospholipid bilayer (Fig. 7B). FakB(FA) then binds FakA, which phosphorylates the FakB-bound FA. FakB(FA~P) then delivers the acyl-phosphate to either PlsY or PlsX for phospholipid or FA synthesis (31, 32), and the empty FakB returns to the membrane to pick up another FA. FA kinase is also essential for the activation of exogenous FA for incorporation into membrane phospholipids (29). However, this pathway has a limited bandwidth, and exogenous FA can be introduced to the growth medium at levels that easily overwhelm the capacity of *S. aureus* phospholipid biosynthesis, leading to repression of SaeRS-dependent virulence factor transcription in wild-type bacteria (25). FA synthesis is an energy-intensive process (43), and both of these functions of FA kinase represent pathways devoted to the efficient utilization of resources. Another role of FA kinase is to prevent the accumulation of membrane-disrupting, nonesterified FA, which will introduce additional negative charge and impart curvature elastic stress that alters the biophysical properties of the phospholipid bilayer (44). The origin of the FA pool in *S. aureus* is unknown. *S. aureus* contains multiple genes with predicted esterase, lipase, and lysophospholipase activities (45), and their hydrolytic activities may be responsible for phospholipid turnover. Another source may be the hydrolysis of the unstable acyl-phosphate (FA~P) intermediate, which is known to occur in cells where PlsY activity is blocked (46). Regardless of the metabolic process that generates FA in *S. aureus*, FA kinase recycles FA into membrane phospholipids and maintains a low level of nonesterified FA in the cell.

The biochemical mechanism that explains FA inhibition of SaeRS signaling remains to be elucidated (Fig. 7B). The experimental results linking the control of SaeS activity to the conformation of the membrane anchor domain (26, 27) suggest several possibilities for FA regulation. One idea is that SaeS responds to the net overall negative charge of the bilayer imparted by the presence of a higher mole fraction of FA. FA are also known to introduce curvature elastic stress into phospholipid bilayers (44), and this alteration in the membrane biophysical properties may alter the conformation of the SaeS membrane anchor domain. FA may act as a classical ligand for SaeS binding to a site on the transmembrane domain to stabilize its inactive conformation. Furthermore, the Sae system has the additional complexity of involving the accessory proteins SaeP and SaeQ, which modify SaeS signaling (47), and may potentially be the FA-interacting component in the system. Deciding between these possibilities will require biophysical experiments to determine how phospholipid bilayer composition impacts the conformation of the SaeS transmembrane helices and/or its interactions with accessory proteins. It is clear that not all regulation of SaeS activity is FA dependent. Defensin activation of SaeS occurs in Δ*fakA* strains (37), illustrating FA-independent control of SaeS activity in this circumstance. Unlike environmental signals that impact SaeRS activity (22, 23, 37, 48, 49), FA are normal constituents of the cellular lipidome whose concentrations create a cellular set point for SaeS signaling that is controlled by the activity of FA kinase. The FA kinase two-component system is conserved in Gram-positive bacteria (29), and it seems likely that its biochemical roles in exogenous FA activation and cellular lipid homeostasis are common themes in this group of bacteria. FA kinase clearly has these housekeeping functions, but whether its activity is regulated to control FA levels and hence SaeS activity is an open question. More work will be needed to determine if the role of FA as cellular signaling molecules is more widespread or whether the *S. aureus* SaeRS two-component system is a unique example of this type of regulation.

## MATERIALS AND METHODS

### Materials.

Fatty acid-free BSA, myristic acid, oleic acid, palmitic acid, and 5,5′-dithio-bis-(2-nitrobenzoic acid) (DTNB) were purchased from Sigma-Aldrich (St. Louis, MO). Antibiotics, high-density nickel resin, and IPTG were purchased from GoldBio (St. Louis, MO). [1-^14^C]acetate (55 mCi/mmol) and [1-^14^C]oleate (56.3 mCi/mmol) were purchased from PerkinElmer (Waltham, MA). Bacteria media supplies were purchased from BD Medical Technologies (Franklin Lakes, NJ). All chemicals and solvents were reagent grade or better.

### Growth conditions and strain construction.

Bacteria were grown in Luria (LB) broth. When appropriate, 1.5 μg/ml of erythromycin, 10 μg/ml chloramphenicol, or 100 μg/ml ampicillin was added. *S. aureus* strains were transformed as previously described (50). Strains and plasmids used in this study are listed in Table S1. Strains PDJ50 and PDJ51 were constructed by allelic replacement (51). In brief, to make strain PDJ50, primers were ordered to amplify regions upstream (*saeS*-5-ERI-F and *saeS*-5-Kpn-R) and downstream (*saeS*-3-Kpn-F and *saeS*-3-Sal-R) of *saeS*. These regions were cloned into the pJB38 vector (51). Once confirmed by sequencing, the plasmid was transformed into strain Sa178RI. A single transformant was inoculated into 5 ml of tryptic soy broth with 10 μg/ml chloramphenicol and grown overnight. A portion of this overnight culture was transferred to growth at 43°C for 48 h to initiate recombination. Bacteria were then plated and single colonies selected and grown at 37°C overnight. Overnight cultures were diluted and plated on tryptic soy agar containing anhydrotetracycline to initiate plasmid loss through another round of recombination. Positive colonies were grown on medium with or without chloramphenicol, and those colonies sensitive to the drug were tested by PCR. To construct strain PDJ51, *saeR*-ERI-F and *saeR*-Kpn-R primers were ordered to amplify the region upstream of SaeR and ligated into a plasmid with the downstream sequence used to produce strain PDJ50.

10.1128/mBio.00988-17.2 TABLE S1  Strains and plasmids used in this study. Download TABLE S1, DOCX file, 0.03 MB. Copyright © 2017 Ericson et al. 2017 Ericson et al. This content is distributed under the terms of the Creative Commons Attribution 4.0 International license.

### Plasmid construction and primers.

The CAT promoter plasmid was constructed using the pCM28 plasmid as a backbone with erythromycin resistance. The CAT gene was amplified from plasmid pSK5483 and cut with PstI and SphI before ligation into pCM28. The *saePQRS* promoter was cloned in front of the coding sequence for CAT by using BamHI and KpnI. Primers were designed to mutate the SaeR binding sites, and the QuikChange Lightning site-directed mutagenesis kit (Agilent, Santa Clara, CA) was used according to the manufacturer’s protocol. Genes encoding NgAas and *S. aureus* FakA were cloned into pG164 using restriction sites BamHI and HindIII. NgAas was then transferred from pG164 to pCS119 using SalI and NheI restriction enzymes. Plasmid pCS119 was created by amplifying the *sarAP1* promoter from USA300 using primers *sarAP1*EcoRI F and *sarAP1*NheI R. The PCR product was digested with EcoRI and NheI and ligated into a cut pCM28 plasmid. Minipreps were performed on transformants, and all plasmids were confirmed by sequencing. Primers used in this study are listed in Table S2.

10.1128/mBio.00988-17.3 TABLE S2  Primers used in this study. Download TABLE S2, DOCX file, 0.02 MB. Copyright © 2017 Ericson et al. 2017 Ericson et al. This content is distributed under the terms of the Creative Commons Attribution 4.0 International license.

### Protein purification.

FakA and FakB proteins were expressed and purified as previously described (29, 30). SaeR, SaeS, and SaeS(K252A R298A) were synthesized with an amino-terminal His tag from Invitrogen (Carlsbad, CA). First, the genes were inserted into the TOPO Zero Blunt plasmid from Invitrogen and inserts were confirmed by sequencing. Genes were excised using BamHI and NdeI and ligated into a cut pET28a plasmid. After sequence confirmation, the pET28a constructs were transformed into *Escherichia coli* BL21 cells. Two liters of cells was grown to an optical density at 600 nm (OD_600_) of 1 before adding 1 mM IPTG for overnight induction at 16°C. Cells were harvested by centrifugation at 4,000 relative centrifugal force (rcf) for 10 min. Cell pellets were resuspended in 25 ml of lysis buffer (20 mM Tris-HCl [pH 7.9], 0.5 M NaCl, protease inhibitor, lysozyme) and lysed with a microfluidizer. Centrifugation at 36,000 rcf for 1 h removed debris. Supernatant was added to a 4-ml Ni-nitrilotriacetic acid column that had been washed, charged with 50 mM NiSO_4_, and equilibrated with MCAC-0 (20 mM Tris-HCl [pH 7.9], 0.5 M NaCl, 10% glycerol). After the supernatant was run over the column, it was washed with 50 ml of MCAC three times with an increasing concentration of imidazole (0 mM, 25 mM, 50 mM) before the proteins were eluted in a buffer containing 400 mM imidazole. Proteins were checked on an SDS-PAGE gel to confirm correct size, concentrated, and purified on a Superdex 200 16/600 column (GE Healthcare, Little Chalfont, United Kingdom), using 20 mM Tris-HCl (pH 7.9), 0.5 M NaCl as the collection buffer.

### Phosphorylation assay.

Phosphorylation assays of SaeS and SaeR with FakAB were performed in buffer with 10 mM Tris-HCl (pH 7.5), 5 mM MgCl_2_, 50 mM KCl, and 0.1 μCi of [γ-^32^P]ATP in a final volume of 20 μl. SaeR and SaeSK252A R298A were held constant at 5 μM when being tested for phosphorylation. SaeS, FakA, and/or FakB were added at increasing concentrations of 1, 2, 5, and 10 μM when appropriate. FA was added to a final concentration of 10, 20, 40, or 80 μM when appropriate. Phosphorylation reaction mixtures were incubated for 30 min at room temperature before being fractionated on a 10% SDS-polyacrylamide gel. Gels were stained with Coomassie brilliant blue dye, destained, and dried for 1 h before exposure on a phosphor screen from GE.

### FA kinase assays.

FA kinase assays were performed as previously described (29). Briefly, reactions were run in mixtures containing 0.1 M Tris-HCl (pH 7.5), 20 mM MgCl_2_, 10 mM ATP, 1% Triton X-100, 20 μM [1-^14^C]oleate, 1 μM FakB2, and 0.1, 0.25, 0.5, 1, or 2 μM FakA in a final volume of 60 μl. Addition of FakA initiated the reactions, and the mixtures were incubated at 37°C for 20 min. Forty microliters of each reaction mixture was spotted onto DE81 disks, which were washed for 30 min, three times in ethanol with 1% acetic acid. Disks were dried and scintillation was quantified on a Beckman counter (Brea, CA).

### Metabolic labeling.

Overnight cultures were diluted to an OD_600_ of 0.05 in 10 ml of LB broth, and 50 μCi of [^14^C]acetate was added. Cultures were grown for 3 h before being harvested. Bligh-Dyer lipid extraction was used on the cells and when appropriate on the medium (52). In brief, cell pellets were resuspended in 1 ml of water. Next, 2.4 ml of methanol with 2% acetic acid and 1 ml of chloroform were added. Mixtures were incubated at room temperature for 30 min, 1.5 ml of chloroform and 1.2 ml of water were added, and samples were vortexed and centrifuged for 10 min. The lower phase was removed and blown down under nitrogen. Because 10 ml of medium was extracted for each experimental condition, volumes added for lipid extraction from medium were 10 times higher. Lipid extractions of media were performed in a separatory funnel with the bottom organic layer easily removed. The lipid fraction was then concentrated under nitrogen before resuspension in a 1:1 chloroform-methanol mixture. Scintillation counts in 5 μl of sample were determined, and equal counts of each sample were loaded onto a Silica G plate from Analtech (purchased from Sigma), which was then developed with hexane-diethyl ether-acetic acid (80/20/1 [vol/vol/vol]). Plates were allowed to dry and then exposed overnight to a phosphor screen.

### Mass spectrometry.

Cells were grown to an OD_600_ between 2 and 3 in all experiments to minimize any variations in FA content as a function of growth phase. Cells (10 ml) were harvested and lipids were extracted using the Bligh-Dyer materials and methods (52). At the beginning of the lipid extraction, D4-16:0 (4 nmol) was added to each sample to serve as an internal standard for quantification of FA levels in the sample. Lipid extracts were dried completely under N_2_ gas and resuspended in 400 μl of chloroform-methanol (1:1). Free fatty acids were analyzed using a Shimadzu Prominence ultrafast liquid chromatograph attached to a Sciex QTrap 4500 apparatus equipped with a Turbo V ion source (Sciex, Framingham, MA). Samples were injected onto an Acquity UPLC BEH HILIC column (1.7 μm, 2.1 by 150 mm; Waters, Milford, MA), using a flow rate of 0.2 ml/min. Solvent A was 100% acetonitrile, and solvent B was 15 mM ammonium acetate (pH 3). The high-performance liquid chromatography program was the following: 0 to 2 min isocratic at 4% B; 2 to 20 min linear gradient to 80% B; 20 to 23 min isocratic at 80% B; 23 to 25 min linear gradient to 4% B; 25 to 30 min isocratic with 4% B. The QTrap 4500 was operated in the negative mode, and the ion source parameters were the following: ion spray voltage, −4,500 V; curtain gas, 25 lb/in^2^; temperature, 350°C; ion source gas 1, 40 lb/in^2^; ion source gas 2, 60 lb/in^2^; declustering potential, −40 V. The system was controlled and analyzed by using Analyst software (Sciex). Cell numbers were determined by making serial dilutions of a culture and counting the colonies.

### Chloramphenicol acetyltransferase assay.

For CAT assays, overnight cultures were diluted in 10-ml volumes to an OD_600_ of 0.05 in LB. When appropriate, BSA was added. After growth for 3 to 4 h, cells were harvested and resuspended in 100 μl of a lysis buffer of 25 mM Tris-HCl, 10 mM EDTA, protease inhibitor, and DNase I. Six microliters of 5 mg/ml lysostaphin was added to each sample, and then the mixtures were incubated at room temperature for 20 min. Lysed cells were centrifuged at 14,000 rpm for 30 min, and 5 μl of supernatant was used for the CAT assay. The CAT assay reaction buffer contained 100 mM Tris, 400 mM DTNB, and 1 μM acetyl coenzyme A. Ninety-three microliters of this mixture was added to the supernatant. Lastly, 2 μl of a 1.6-mg/ml chloramphenicol solution was added to each reaction mixture. The production of TNB from these reactions was quantified using a SpectraMax machine (Molecular Devices, Sunnyvale, CA) in kinetic mode, which read the change in absorbance at 412 nm every 10 s. To convert the rate of the change of absorbance to millimoles of TNB produced, the change was divided by 13.6, as described previously (53). Rates of TNB conversion were normalized by running a bicinconinic acid (BCA) assay for determination of protein concentration. Before cells were harvested for the CAT assay, 200 μl of each sample was collected and cells were harvested and then resuspended in 100 μl of 100 mM Tris-HCl (pH 7.5), 0.1% SDS, and 0.1% Triton X-100. Mixtures were incubated on ice for 20 min and then at 100°C for 30 min. Samples were then centrifuged at 14,000 rpm for 15 min, and 25 μl was added to the working reagent made from a BCA protein kit from Thermo Fisher (Waltham, MA). BSA was used as a standard to quantify protein amounts. After the sample and working reagent were mixed, they were incubated at 37°C for 30 min. The protein amount was measured using a SpectraMax reader based on absorption at 562 nm. The protein determinations correlated with the cell numbers, and normalization of CAT activity based on the OD_600_ gave the same relative results.

### Quantitative real-time PCR.

For qRT-PCR, *S. aureus* strains USA300, JLB2 (Δ*fakA*), JLB31 (*ΔfakB1&2*), Sa178R1, Sa178R1/pNgAas, and Sa178R1/pFakA were grown to an OD_600_ of 2. For BSA experiments, JLB2 (Δ*fakA*) cells were grown with or without FA-free BSA, and for FA experiments strains were grown to an OD_600_ of 0.5 before FA was added to BSA to a final concentration of 500 μM. RNA was isolated with an RNAqueous purification kit (Ambion, Austin, TX) according to the manufacturer’s specifications. Purified RNA was then mixed with 0.5 vol of LiCl precipitation solution (Ambion) and left at −20°C for 30 min. Turbo DNase (Ambion) was added to the precipitated RNA to a final concentration of 1 U of DNase per 5 μg of RNA. This mixture was incubated at 37°C for 30 min. Integrity of the RNA was assessed by agarose gel electrophoresis and by using an Agilent Technologies 2100 Bioanlyzer Lab-on-a-Chip system, before the RNA was used in the qRT-PCR. Primers for *ehp*, *efb*, and *saeP* have been previously reported (22). Each 20-μl RT reaction mixture contained 500 ng of RNA, 12.5 ng/μl of random hexamers (Invitrogen), 10 mM deoxynucleoside triphosphates (Sigma), 40 U of RNaseout (Invitrogen), and 10 U/μl of SuperScript II reverse transcriptase (Invitrogen). After the RT reaction, 2-μl aliquots of the cDNA product were added to an RT-PCR reaction mixture with 1× TaqMan universal PCR master mix (Applied Biosystems, Foster City, CA), 660 nM of each forward and reverse primer, and 150 nM of probe. Specific products were detected on an ABI Prism 7700 sequence detection system (Applied Biosystems) using the following conditions: 50°C for 2 min, 95°C for 10 min, 95°C for 15 s for 40 cycles, and 60°C for 1 min. Samples were processed in 96-well plates. Three RT-PCRs were performed on each cDNA sample. cDNA was prepared from separate cultures for each biological condition. No-template mixtures and reaction mixtures without reverse transcriptase were run as negative controls. Real-time values were evaluated using the threshold cycle (*C*_*T*_) method, with genes being normalized to a *gmk* calibrator (54).

### FA removal by BSA.

Overnight cultures of strains USA300, JLB2 (Δ*fakA*), and PDJ50 (Δ*saeS*) were diluted to an OD_600_ of 0.05 in 10 ml of LB medium in the absence or presence of 10 mg/ml FA-free BSA. For metabolic labeling, cells were grown with FA-free BSA for 3 h before cell pellets were harvested and lipid extraction was performed. For CAT assays, bacterial cultures were grown to an OD_600_ of 2 and used for further analysis.

### Hemolysis assay.

For the hemolysis assay, overnight cultures were diluted to an OD_600_ of 0.05 and allowed to grow to mid-log phase. Upon reaching mid-log growth, 1 μl of the culture was spotted onto an LB agar plate supplemented with 3% rabbit blood. Plates were incubated at 37°C overnight, after which zones of clearance were examined.

### Statistics.

Data sets were compared using the two-tailed Student *t* test, and statistical significance is indicated in the figures (*, *P* < 0.05; **, *P* < 0.01; ***, *P* < 0.001).

### Availability of data.

Strain descriptions and data that support the findings of this study are available from the corresponding author upon request. 
