# Supplementary figures and images for: Role of Fatty Acid Kinase in Cellular Lipid Homeostasis and SaeRS-Dependent Virulence Factor Expression in Staphylococcus aureus
Source: mBio. 2017 Aug 1;8(4):e00988-17. doi: 10.1128/mBio.00988-17 (PMC5539427; doi:10.1128/mBio.00988-17)

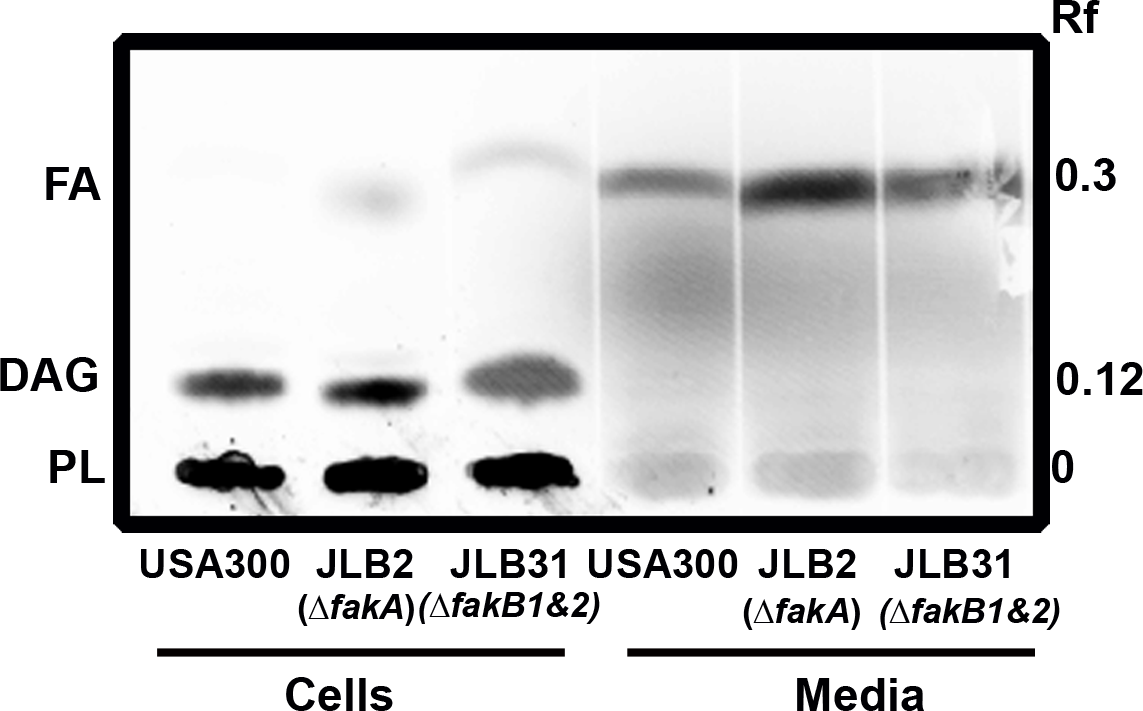

Supplement: FIG S1 [file mbo004173416sf1.tif]
