# Supplementary material for: Role of Fatty Acid Kinase in Cellular Lipid Homeostasis and SaeRS-Dependent Virulence Factor Expression in Staphylococcus aureus
Source: mBio. 2017 Aug 1;8(4):e00988-17. doi: 10.1128/mBio.00988-17 (PMC5539427; doi:10.1128/mBio.00988-17)
Supplement: TABLE S1 [file mbo004173416st1.docx]

Table S1: **Strains and plasmids**

| Strains | | |
| --- | --- | --- |
| *Strain Name* | *Genotype* | *Source* |
| AH1263 | USA300 CA-MRSA | 1 |
| JLB2 | USA300Δ*fakA* | 2 |
| JLB31 | USA300Δ*fakB1&2* | 3 |
| PDJ50 | USA300*ΔsaeS* | This study |
| PDJ51 | USA300*ΔsaeRS* | This study |
| Sa178RI | CYL316 with T7 RNA Polymerase | 4 |
| PDJ42 | Sa178RIΔ*fakA* | 5 |
| PDJ43 | Sa178RIΔ*fakB1&2* | 5 |
| Newman | SaeS^L18P^ | 6 |
| Plasmids |  |  |
| *Plasmid Name* | *Description/Drug Resistance* | *Source* |
| pG164 | Shuttle vector for T7 based protein expression/Chl | 4 |
| pPJ497 | pG164FakA/Chl | This study |
| pJD001 | pG164NgAas/Chl | This study |
| pCS119 | pCM28SarAP1promoter/Chl | This study |
| pJLB165 | pCM28FakA/Chl | 2 |
| pPJ507 | pCM28NgAas/Chl | This study |
| pCS125 | PCM28_CAT assay/Erm | This study |
| pKM264 | pCM28_CATsaePQRS/Erm | This study |
| pKM267 | pCM28_CATsaePQRSm1/Erm | This study |
| pKM268 | pCM28_CATsaePQRSm2/Erm | This study |
| pET15b | Protein Expression/Amp | Novagen |
| pET28a | Protein Expression/Km | Novagen |
| pJLB11 | pET28a-FakA/Amp | 3 |
| pCS107 | pET15b-FakB2/Amp | 3 |
| pCS104 | pET28a-SaeR/Amp | This study |
| pME001 | pET28a-SaeS/Amp | This study |
| pME003 | pET28a-SaeSK252AR298A/Amp | This study |
| pJB38 | Allelic Replacement/Chl | 7 |

1 Kreiswirth, B. N. *et al.* The toxic shock syndrome exotoxin structural gene is not detectably transmitted by a prophage. *Nature* **305**, 709-712, (1983).

2 Bose, J. L., Daly, S. M., Hall, R. R. & Bayles, K. W. Indentification of the *vfrAB* operon in *Staphylococcus aureus*: a novel virulence factor regulatory locus. *Infect. Immun* **82**, 1813-1822, (2014).

3 Parsons, J. B. *et al.* Identification of a two-component fatty acid kinase responsible for host fatty acid incorporation by *Staphylococcus aureus*. *Proc. Natl. Acad. Sci. U. S. A* **111**, 10532-10537, (2014).

4 D'Elia, M. A. *et al.* Lesions in teichoic acid biosynthesis in *Staphylococcus aureus* lead to a lethal gain of function in the otherwise dispensable pathway. *J. Bacteriol* **188**, 4183-4189, (2006).

5 Broussard, T. C. *et al.* Biochemical roles for conserved residues in the bacterial fatty acid binding protein family. *J. Biol. Chem*, (2016).

6 Parsons, J. B. *et al.* Perturbation of *Staphylococcus aureus* gene expression by the enoyl-acyl carrier protein reductase inhibitor AFN-1252. *Antimicrob. Agents Chemother.* **57**, 2182-2190, (2013).

7 Bose, J. L., Fey, P. D. & Bayles, K. W. Genetic tools to enhance the study of gene function and regulation in *Staphylococcus aureus*. *Appl. Environ. Microbiol.* **79**, 2218-2224, (2013).
