# Supplementary material for: Role of Fatty Acid Kinase in Cellular Lipid Homeostasis and SaeRS-Dependent Virulence Factor Expression in Staphylococcus aureus
Source: mBio. 2017 Aug 1;8(4):e00988-17. doi: 10.1128/mBio.00988-17 (PMC5539427; doi:10.1128/mBio.00988-17)
Supplement: TABLE S2 [file mbo004173416st2.docx]

Table S2: **Primers**

| Primer | Sequence (5′-3′) |
| --- | --- |
| *saePQRS* long for | GGATCCAATTATTTTCATTTTCAAATTATTCTTTCTTC |
| *saePQRS* long rev | GTTGTGATAACAGCACCAGCTGC |
| *saePQRS* mut1 for | CGATATTTAAACCAAGTTGGGAATTAGTTAATGGCATATTATTTGC |
| *saePQRS* mut1 rev | GCAAATAATATGCCATTAACTAATTCCCAACTTCGTTTAAATATCG |
| *saePQRS* mut 2 for | CGATATTTAAAACGAAGTTAAGAATTAGGGAATGGCATATTATTTGC |
| *saePQRS* mut2 rev | GCAAATAATATGCCATTCCCTAATTCTTAACTTCGTTTAAATATCG |
| *saeP* RT for | CGGTGAAACTGTTGAAGGTAAAGCTGA |
| *saeP* RT rev | TTAGCGCCGCCGAAGATGACG |
| *ehp* RT for | ACGGTATCAACGTTTGCCGGTGA |
| *ehp* RT rev | GCTCTTTGTGCTTTACGGTGTGTTGC |
| *efb* RT for | AACAGCAGATGCGAGCGAAGG |
| *efb* RT rev | TGCATCAGTTTTCGCTGCTGGT |
| *saeR-*ERI-F | GTACCAGAATTCGTGAAACTGTTGAAGGTAAAGCTG |
| *saeR-*Kpn-R | ATACACGGTACCATCATCCACGATCAGTAAGTG |
| *saeS*-5-ERI-F | TCATATGAATTCGGATTTACGCTGAAAGGTTTAG |
| *saeS*-5-Kpn-R | TATCTAGGTACCACCATTATCGGCTCCTTTC |
| *saeS*-3-Kpn-F | TGATCAGGTACCAGGAACTACGATGACTGTAAC |
| *saeS*-3-Sal-R | ATCTAAGTCGACAAGATAGTAGTGTGAAAGGC |
| *sarAP1* EcoRI F | ACTAGAATTCCTGATATTTTTGACTAAACCAAATG |
| *sarAP1* NheI R  *NgAas* BamH1 F  *NgAas* EcoR1 R | ATACGCTAGCGTTAATTATAACTAATTAAAAATGAGAAG  ATATGGATCCATGAATCGTACCTACGCCAACTTTTATG  GATTACGAATTCTTAGTGGTGATGGTGATGATGGTTACC |
